# Efficiency in health care: connecting economic evaluations with implementation objectives

**DOI:** 10.1186/s43058-025-00763-4

**Published:** 2025-07-26

**Authors:** Todd H. Wagner, Ties Hoomans, Ramzi G. Salloum, Douglas E. Levy

**Affiliations:** 1 Health Economics Resouce Center, US Department of Veterans Affairs, Menlo Park, USA; 2 Department of Surgery, Stanford University, Stanford, CA USA; 3 Care Policy and Evaluation Centre (CPEC) at the London School of Economics and Political Science, London, England; 4 Division Director for Implementation Science in the Department of Health Outcomes & Biomedical Informatics, University of Florida College of Medicine, Gainesville, USA; 5 Harvard Medical School and the Mongan Institute Health Policy Research Center at Massachusetts General Hospital, Boston, USA

**Keywords:** Efficiency, Productivity, Waste, Costs, Economies of scale, Behavioral economics

## Abstract

**Introduction:**

Economic evaluations are helpful for efficient resource use. This paper aims to clarify the relationship between economic evaluation methods and two types of health care efficiency, aiding implementation scientists in selecting the appropriate approach for their research.

**Methods:**

We clarify the connection between cost-effectiveness analysis (CEA) and allocative efficiency, and explain how budget impact analysis (BIA) more closely connects with productive efficiency. We also discuss other methods that researchers can use to analyze an organization's productive efficiency, given increasing pressure for health care organizations to be efficient.

**Results:**

Allocative efficiency seeks to maximize social welfare through optimal resource distribution. Productive efficiency focuses on an organization’s ability to maximize its output given its resource constraints. CEA, particularly when incorporating a societal perspective, assesses allocative efficiency. BIA, which often has a short time horizon and more focused perspective, assesses productive efficiency. When organizational leaders ask implementation scientists for an economic evaluation, it is important to determine whether they want a CEA or a BIA, given they answer different questions, often employing different methods. We also present other methods for measuring efficiency and causes of inefficiency stemming from fixed costs, scale, scope, regulations, labor, and decision-making.

**Conclusions:**

Implementation scientists must recognize that CEA and BIA serve distinct purposes and are not interchangeable. Choosing the right economic evaluation tool is crucial for answering specific research questions and building research teams. Future implementation work will also need to measure efficiency so that it is sustainable.

Contributions to the literature

- Implementation scientists seek to conduct economic evaluations to support learning health care systems
- Economic evaluations, such as cost-effectiveness analysis and budget impact analysis, address two different aspects of efficiency, thus understanding efficiency is helpful for choosing the right method.
- We highlight how efficiency analysis may help sustain implementation strategies.

## Introduction

Leaders in health care delivery organizations must make decisions and set priorities to improve care and outcomes with limited budgets. Not only do they need to invest in the right services, but they must also consider the resources needed for implementation and practice change. Wise decisions can help make the organization more efficient, while poor choices can create inefficiencies that persist for years. For example, investing in a new diagnostic tool without adequate training and workflow integration may limit its impact and waste resources. Similarly, prematurely scaling a remote patient monitoring program in primary care without addressing barriers to adoption and maintenance may result in low effectiveness and limited reach [1].

To navigate complex decisions and build learning health care systems [2], organizational leaders are increasingly asking implementation scientists to conduct economic evaluations to ensure efficient resource use. There are many types of economic evaluations, and each informs a different aspect of efficiency. Although defining efficiency sounds simple, anyone who ventures into the efficiency literature will be confronted with substantial technical details and jargon, with the term used in different, and sometimes seemingly conflicting contexts [3]. The objective of this paper is to clarify these connections and to help implementation scientists select the right method for their economic evaluation.

## The link between economic evaluations and efficiency

Health care efficiency falls into two main types: allocative efficiency—distributing resources in a way that best achieves societal goals, such as population health or social welfare, and productive efficiency—an organization’s ability to maximize the production of its outputs from available inputs and technology. Allocative efficiency is important for national policy makers, such as the National Health Service in the United Kingdom or the Centers for Medicare and Medicaid Services in the United States, while hospital directors and clinic managers may be more focused on productive efficiency. As we discuss below, allocative and productive efficiency relate to different economic evaluations.

### Allocative efficiency and cost-effectiveness analysis

**Fig. 1:**
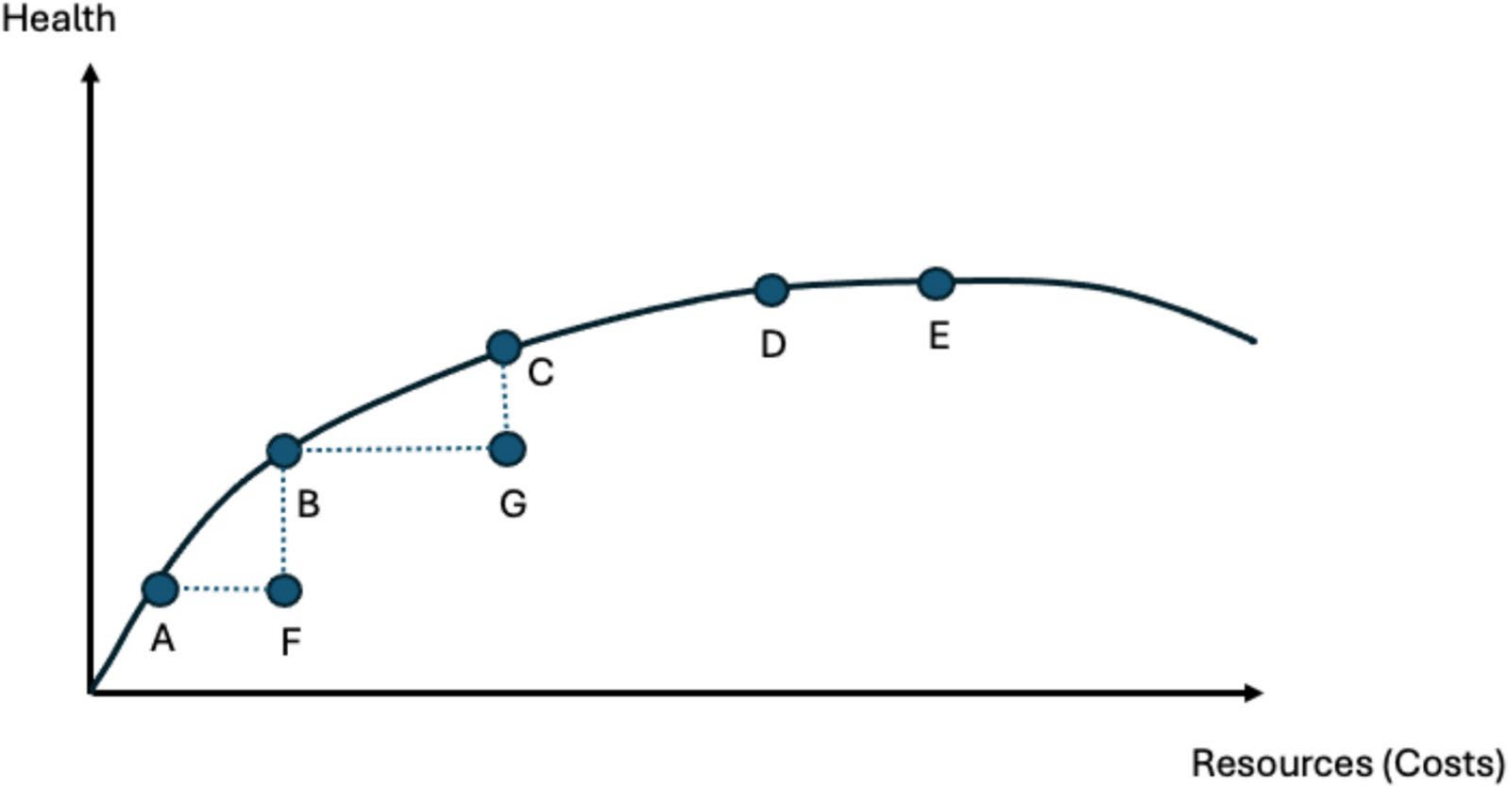
Allocative efficiency and production possibilities frontier. Caption: points A-G represent interventions. The production possibility frontier shows what health could be produced with different interventions. Interventions A-E all improve health, but require more resources. Interventions F and G and not cost-effective compared to alternatives. Health in this context is population health, what economists refer to as social welfare

Cost-effectiveness analysis (CEA) is the best-known tool for conducting economic evaluations in health and medicine. In the years after Weinstein and Stason’s [4] landmark paper published almost fifty years ago, experts recommended conducting CEA from a societal perspective with a long-term time horizon [5, 6]. Although comprehensive societal analyses are rare due to data limitations [7], this approach provides information on whether a new treatment or program efficiently maximizes social welfare [8]. In theory, social welfare encompasses a society’s health, estimated by aggregating quality of life and social well-being across individuals. Efficiency in this context is referred to as allocative efficiency; put simply it seeks to address the question, does the allocation of resources maximize social welfare? Fig. 1, adapted from Chandra et al., [9] shows the relationship between the use of resources invested in health on the X axis and amount of health outputs those resources produce on the Y axis. This figure is referred to as the health care production possibilities frontier. Points A-G all represent different interventions. Interventions A-E all generate gains in health but at an added cost. A cost-effectiveness analysis would show that interventions F and G are not cost-effective, relative to other treatment options. Societies can choose to how much to invest in health care, and higher investments yield more health, but there is a tradeoff because investments in health consume more resources that could be invested elsewhere (e.g., education or roads) to generate social welfare.

On Fig. 1, the Y axis represents health or idealized wellness. We do not have a perfect measure of health. Quality adjusted life years (QALYs) and disability adjusted life years (DALYs) are widely used, but even these methods have challenges and limitations [10, 11]. Production above the curve is not possible, given current technology and medical knowledge. Innovations provide opportunities to produce more health outputs, but often at an additional cost. For example, Chimeric Antigen Receptor T-Cell therapy has provided a new way to treat acute lymphoblastic leukemia, but the cost of the drug can exceed USD $450,000. Not surprisingly, many ask, “is the new innovation worth it?” CEA uses the incremental cost-effectiveness ratio to compute the additional gains in health relative to the additional resources required to achieve them.

At any point the production possibilities frontier shown on Fig. 1, the slope of the curve represents the rate of spending relative to the outputs produced. Moving from points A to B requires about the same resources as moving from D to E, but moving from D to E produces substantially fewer gains in health than moving from A to B. The slope at point E is nearly zero. This is referred to as”the flat of the curve.” [12] In cancer screening, low-cost strategies such as patient reminders, may result in 70–80% of the population getting screened. But more expensive strategies are usually needed to improve screening rates further. It is also possible to overinvest in health, as shown on the far right of the curve, a phenomenon Grady and Redberg [13] refer to as when “Less is More.” While most experts would recommend not investing in technologies or programs on the flat of the curve, each government can set its own willingness to pay for gains in health. This is the willingness to pay threshold that commonly appears in CEA papers [14].

CEAs that take a societal perspective (i.e. to assess the health of nations) may not answer questions posed by health care organizations. Individual organizations generally have narrower interests within their programmatic priorities and budgetary constraints. The Second Panel on Cost-Effectiveness in Health and Medicine acknowledged the need for CEA models with shorter time horizons and more limited perspectives [15]. Such modifications may be appealing to organizational decision makers, but as we discuss next, these models may not be measuring allocative efficiency and instead they may be measuring productive efficiency.

### Productive efficiency and budget impact analysis

Health care organizations provide health care services to patients by investing in staff, supplies, space, and equipment— what economist generically refer to as labor and capital. Each organization has its own priorities and objectives, whether that is maximizing profit or providing as many health services as possible [16]. Organizations are efficient when they maximize the quantity of outputs they produce given the resources or inputs used, along with the available technology and knowledge [17]. No health care organization strives to be inefficient because it implies that its production process is not as good as it could be, and the organization has the potential to produce more output with the same inputs. Efficiency, when used in this context, is referred to as productive or technical efficiency.

**Fig. 2:**
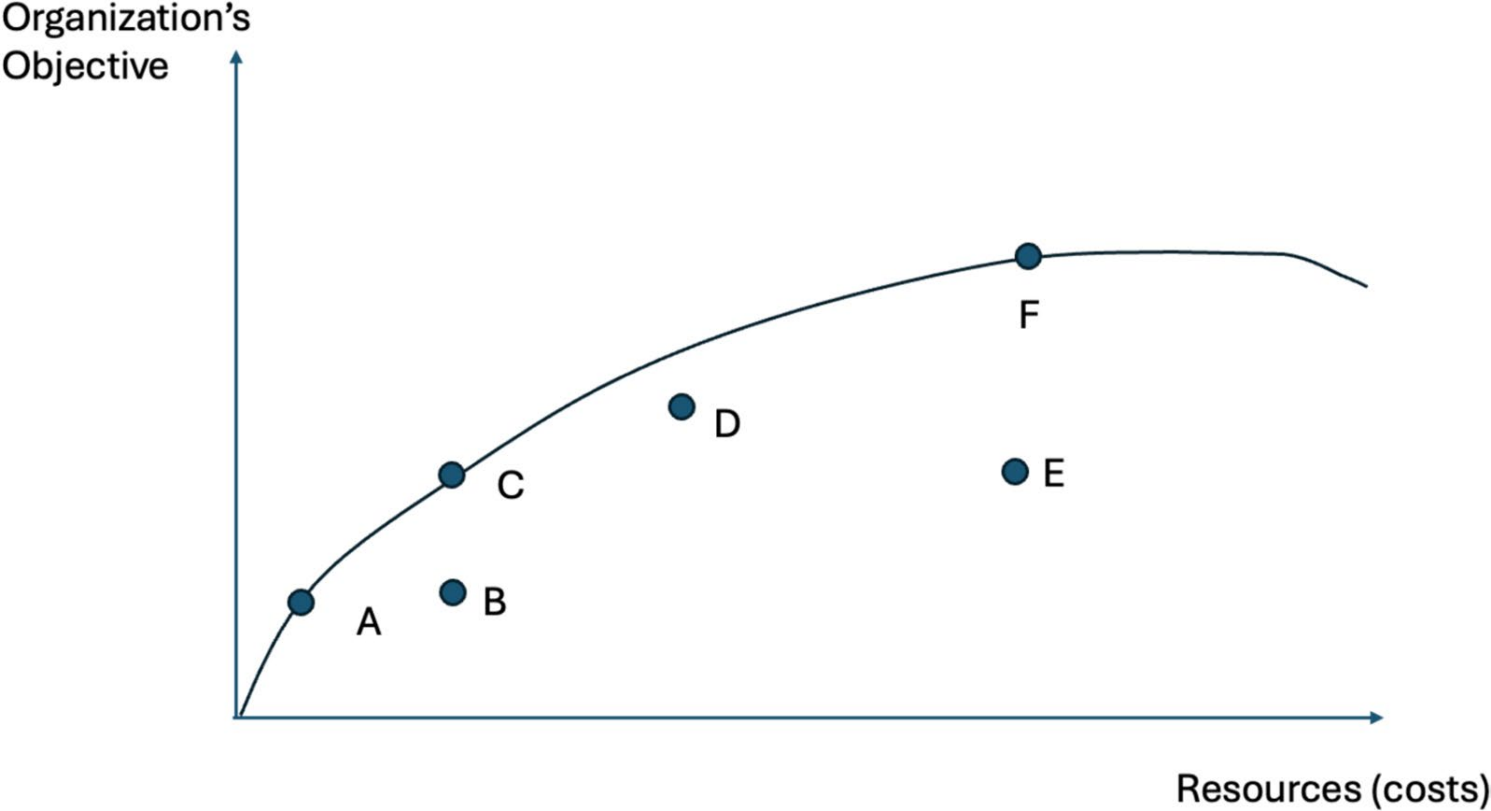
Productive efficiency. Caption: points A-F are health care organizations. The production possibility frontier shows the health that different organizations are producing relative what could be produced if they were productively efficient. The Y axis is health as measured by the organization’s objective function

Again, we can use the production possibilities frontier to represent productive efficiency. Figure 2 looks similar in most regards to Fig. 1, with some important changes. First, the Y axis has changed from social welfare to the organization’s specific objective(s). Rarely are individual organizations trying to maximize the health of society. Next, each point in Fig. 2represents an organization that has allocated its resources on staff, facilities, equipment, and supplies to produce outputs [17]. An organization that invests a greater share of its resources into staff will have fewer resources to allocate to the other inputs. There is no single allocation rule that works equally well for every organization. Primary care clinics generally devote a greater share of their resources to labor, whereas hospitals generally invest a greater proportion of their resources into capital, such as scanners, ventilators, or surgical suites [18]. Finally, we introduce inefficient organizations. Efficient organizations (points A, C and F) are producing on the production possibilities frontier (see Fig. 2) [17]. Conversely, inefficient organizations are producing “off the frontier”; organizations represented by B, D, and E could produce more products and services with the same inputs. Efficient organizations can create goods and services that have lower costs than inefficient organizations, all else being equal. Budget impact analysis (BIA) is a common economic evaluation that can help an organization become more productively efficient [19–22].

### Connecting allocative efficiency and productive efficiency

Organizational leaders are motivated to optimize their organization’s productive efficiency because it affects outcomes they care about. However, their decisions may not be maximizing social welfare. In perfectly competitive markets, productive and allocative efficiency are linked through what Adam Smith referred to as the invisible hand of the market. Perfectly competitive markets are driven by consumers. Organizations respond by providing those services. Thus, perfectly competitive markets provide signals to organizations on what they should produce to maximize social welfare [23]. In a competitive market, this process happens continuously over time. Thus, organizations must continually strive to be efficient [24].

The restaurant industry in most cities, such as New York, is one example where there are tight connections between productive and allocative efficiency. Consumers are well informed through online reviews, and there are few barriers for a restaurant to enter or exit the market. Consumers can easily judge value and they stop going to restaurants where the cost/quality tradeoff is poor, relative to the competition. Thus, implementation science is not common in perfectly competitive markets, like restaurants in most cities, where restaurants that fail to respond to the market signals go out of business.

It is well-established, however, that health care markets are stubborn exceptions to perfect competition [25]. Without a well-functioning market, the tight connection between productive efficiency and allocative efficiency is lost. A health care organization can minimize its costs of production, but still produce too much specialty care or overinvest in technologies that may have little societal value [26]. For example, many hospitals in the US continue to add intensive care beds, even though up to half of beds are not being used by patients needing life sustaining treatment [27, 28].

Unlike restaurants, a health care organization can survive and be sustainable in the long-term, despite being inefficient. To maximize social welfare in health care, two general conditions must hold. First, health care organizations must be productively efficient. Second, health care organizations must be providing services that can maximize allocative efficiency. Implementation scientists can inform and sometimes persuade the organization, by using strategies, to produce services that maximize allocative efficiency.

## Types of economic evaluations

### Cost-effectiveness analysis for allocative efficiency

**Fig. 3:**
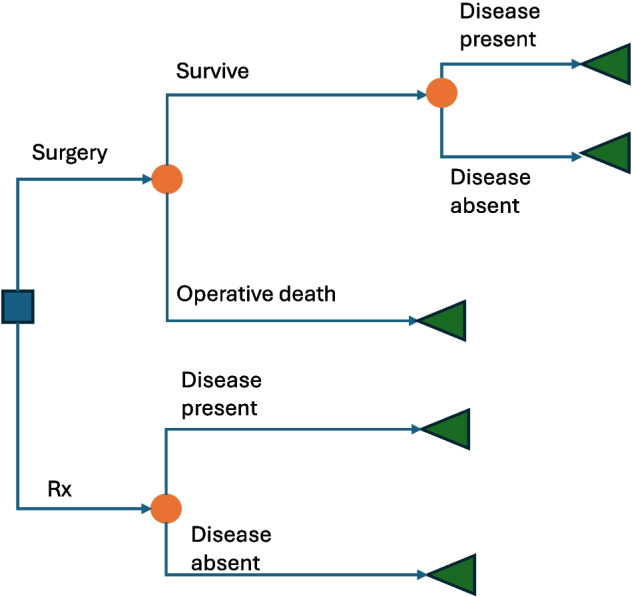
A hypothetical decision tree

Researchers interested in allocative efficiency will gravitate towards a CEA using a decision model. Decision models, such as decision trees, Markov models and discrete event simulations, can be built to inform decision makers about the likely best course of action to maximize allocative efficiency. A simple decision tree is shown in Fig. 3with a hypothetical illness that has two treatment options: medical management or surgery. The decision tree is made up of decision nodes (blue squares), chance nodes (orange circles), and terminal nodes (green triangles). For a CEA, the decision tree should be populated with information on chance node probabilities, treatment costs, and outcomes at the terminal nodes. With this information, the optimal decision can be determined. The gold standard CEA, sometimes referred to as cost-utility analysis, is a decision model that includes lifetime costs and benefits using a societal perspective [5, 6, 15]. These models account for discounting and inflation, based on the time horizon [21]. Societal costs reflect all the resources used by all parties affected by the intervention, not just the healthcare system or a specific payer; this includes treatment costs, patient and caregiver costs, and productivity losses [15]. Some countries use CEA to ensure that resources are efficiently allocated to services that maximize social welfare. For example, CEAs are a key element of policymaking in the United Kingdom through the National Institute for Health and Care Excellence (NICE).

### Budget impact analysis for productive efficiency

Implementation scientists interested in productive efficiency will be less interested in a CEA and more interested in other approaches, where the results can guide short-term decisions. While BIA has grown in popularity, helping an organization optimize its productive efficiency may lead to decisions that are incongruous with allocative efficiency [29]. Ideally a BIA would be accompanied with a CEA to highlight when productive efficiency differs from allocative efficiency. If this is not feasible to do formally (e.g., because of a lack of resources), then investigators should discuss the time horizon used and how the results might change if a different time horizon were used.

### Measuring BIA and productive inefficiency

While a BIA can be estimated using a decision model, examining productive inefficiencies can be accomplished through a range of other methods. Among health economists, it is common to use administrative data with causal inference methods to estimate the effect of a program implementation on economic endpoints. For example, Gujral and colleagues [30] used a difference-in-differences analysis to evaluate a program that distributed tablet computers to patients with mental health needs and limited access to care. Another example is Daniels et al.’s [31] evaluation of a multidisciplinary pain clinic that was implemented in two hospitals. Both examples employ statistical analyses of administrative health care cost and utilization data to estimate budgetary impact.

Economists have also pioneered other approaches to measuring an organization’s productive efficiency using an output maximization or cost minimization models (see Table 1). These models should include implementation efforts and costs, as noted by the i, in Table 1. The output maximization model examines the quantity of outputs that an organization produces relative to the inputs it uses. Estimating the output maximization model frequently requires the use of data envelopment analysis (DEA), a mathematical programming technique that computes relative efficiency scores [32]. Prior and Solà [33] used DEA to examine the efficiency of hospitals, whereas Tran et al. [34] used DEA to measure primary care clinic efficiency. The cost minimization model encapsulates all the organization’s inputs in a single metric: the organization’s operating costs. Analytically, one estimates a regression model known as a “stochastic frontier analysis” in which total operating cost is the dependent variable and the independent variables are the input prices (i.e., the local cost of labor and capital) and the organization’s outputs. A range of regression models have been used to estimate the stochastic frontier from a simple ratio of costs per service to the more flexible translog model [35]. Readers may refer to examples from Romley et al., [36] Gu et al., [37] and Hall [38].

**Table 1:** Two common approaches for measuring productive efficiency

Output maximization	Cost Minimization
Output = f(w, y, r, i, u)	Cost = f(p, o, r, i, u)
Where	Where
Output is the quantity of outputs produced	Cost is the organization’s total operating cost
w is a vector of input labor quantities (e.g., full time equivalent employees)	p is a vector of input prices (i.e., cost of labor and capital) at the local-market level. Most models assume that these prices are set by market forces
y is a vector of input capital quantities (e.g., number of bed days, equipment, and supplies)	o is a vector of outputs, such as the number of outpatient visits and inpatient stays produced
r is a vector of regulatory factors, such as hospital accreditation or internal staff policies	r is a vector of regulatory factors
I is a vector of implementation activities undertaken by an organization for implementation and practice change.^a^	I is a vector of implementation activities undertaken by an organization for implementation and practice change.^a^
u is a vector of patient differences (e.g., age, gender, clinical risk, social risk)	u is a vector of patient differences (e.g., age, gender, clinical risk, social risk)

^a^In markets that are perfectly competitive, the market provides signals to direct quality improvement and implementation activities can be assumed to be zero. However, in health care and education the markets do not provide these signals and implementation costs must be included

### Other economic evaluations

Readers may also encounter return on investment (ROI) analysis and cost–benefit analysis (CBA). A ROI analysis is simply a ratio of benefits relative to investments. A CBA is the difference between the net benefits and costs. In both ROI and CBA, the benefits, including mortality effects, are expressed in monetary terms. Depending on the perspective (what benefits are measured) and the time horizon, ROIs and CBAs can end up looking much like a CEA or a BIA. The perspective and time horizon will determine whether the models are addressing questions that relate to allocative efficiency or productive efficiency.

## Causes of inefficiency

A BIA may identify inefficiencies, but further mechanistic information will be needed if organizational leaders want implementation scientists to help them become more efficient. Some inefficiencies may be easily identified and remedied with little or no external effort. This includes, for example, staffing allocations where some staff are waiting for patients. Conversely, other inefficiencies, such as bottlenecks in patient transfers, may be more difficult to identify, necessitating a comprehensive investigation. If organizational leaders are seeking ways to reduce productive inefficiency, then understanding the underlying mechanisms is critical. Below, we discuss common causes of inefficiencies. These causes might stem from organizational structures, employee decision making, or financing and regulatory factors external to the organization.

### Capital investment and fixed costs

Total costs of running a health care organization include a combination of variable costs, such as labor and supplies, and capital investments, such as equipment and space. These capital investments are often referred to as a fixed cost because once a health care organization purchases a building or a piece of equipment, such as an MRI machine, the cost is fixed for its lifespan (e.g., 8–12 years for an MRI machine). If the organization is not using the MRI machine at full capacity, it can sell it and then invest the money in a more productive capacity. Major investments are often done with projections, but inefficiencies arise when actual service use and costs differ from projected use and costs. To reduce inefficiencies due to excessive fixed costs, Adang and Wensing [29] proposed a fixed cost checklist combined with education so that organizational leaders could make informed decisions for the short and long term. Inefficiencies associated with fixed costs typically do not resolve on their own because it takes effort, and sometimes money, to convert the fixed asset into a variable cost. In the MRI example, the health care organization might seek to reduce costs by selling the MRI machine’s excess capacity to other health care organizations, or start imaging patients who would not necessarily benefit from the MRI (i.e., provider-induced demand [39, 40]). Inefficiencies from fixed costs can be resolved over time. Evaluations that take a long-term time horizon, can estimate costs, assuming that these inefficiencies are resolved. However, in evaluations with a short-term time horizon, which is more common in implementation science, two questions arise: what is the best strategy in the short term given the variable costs, and next, what is the best course of action in the long term when the fixed costs are variable. Wagner [18] gives an example of inefficiencies in ICUs, noting that in the short term, the best strategy is to minimize variable costs, even if ICU beds remain unused. The best strategy in the long term is then to convert the unused beds into more productive capacity.

### Scale and scope

Inefficiencies may be linked to organizational size. Small health care organizations often encounter inefficiencies because they lack the economies of scale achieved by larger organizations. Efficiency gains from economies of scale happen when expanding the scale of production reduces the average unit cost. This may occur, for example, when two small clinics merge, thus combining their administrative and human resources costs, and the combined clinic is less expensive to operate than the combined cost of the two individual clinics. Alternatively, small institutions may improve efficiency by purchasing certain services externally rather than providing them internally. Another commonly described efficiency is economies of scope. Economies of scope arise when a health care organizations conducts a re-organization and the result is a decrease in the total cost of operation [41]. In health care, creating multispecialty group practices has been linked with breaking down “silos” and creating efficiencies [42].

### Rules and regulations

Organizations must adhere to external regulations, but they can also create their own internal rules. Separating these two related concepts is important. In terms of productive efficiency, external regulations increase the organization’s cost of production relative to no regulations (see for example, Keeler and Sing [43]). In terms of allocative efficiency, regulations may enhance social welfare even if they decrease an organization’s productive efficiency. For example, imagine that two health care organizations sought to increase their productive efficiency by merging and gaining economies of scale. However, provider consolidation has been linked to higher prices because it reduces the competition across providers [44–46]. Therefore regulations preventing the merger may improve allocative efficiency while hindering productive efficiency [47, 48]. Assessing those tradeoffs are often the focus of government regulators, like the Federal Trade Commission.

Researchers often assume regulations are fixed. Erhun and colleagues [49] compared the costs of heart surgery in the US relative to India. They noted that major savings could be possible if U.S. health care providers reconsidered or de-implemented many of the regulations that make care in the US so expensive relative to India. Prime examples would be regulations that no longer improve allocative efficiency (add societal value). An example is the change in U.S. Food and Drug Administration’s regulations affecting hearing aids in which the agency determined consumers seeking hearing aids can buy them over the counter without needing an audiology exam. Researchers should consider the possible effects of changing or de-implementing policies and regulations that do not improve allocative efficiency. While public policies may be unlikely to change without considerable efforts through voting, lobbying, and other avenues of political persuasion, changing regulations may be appropriate implementation targets. Organizational policies and rules can also cause inefficiencies, but they may be more amenable to change. Recent research examined inefficiencies from outdated pre-operative testing requirements [50]. Educating organizational leaders about the deleterious effects of low-value requirements may be effective at removing them.

### Labor

Inefficiencies sometimes result from an organization’s suboptimal mix of labor inputs (e.g., doctor, nurse, nursing assistant). For example, organizations might experience inefficiencies when choosing among different types of labor (e.g., advance practice nurses and doctors) [51], or in how they deploy staff. Process maps can help identify staffing bottlenecks and inefficiencies. For example, Dilts and colleagues [52] created process maps to understand the efficiency of initiating clinical trials at their research facility. They mapped the actors, actions and time involved in the process, mirroring the Action, Actor, Context, Target, Time (AACTT) model described by Presseau and colleagues [53]. They used these data in collaboration with leadership to reduce delays in the process. The process maps highlighted which actions were creating bottlenecks and delays in the process. Fernandez and colleagues [54] describe a six-step approach for using process maps to identify targets for improvement and potential strategies that might effect change. Labor inefficiencies may also stem from patient no-shows which reduces productivity, which has been mitigated with patient reminders, overbooking, or no-show fees [55, 56].

### Employee / practitioner decision making

Employee decisions can significantly impact efficiency. Researchers have analyzed decisions over employees’ shifts, noting how the decisions change when faced with a constraint. Freedman and colleagues [57] found that under unexpected time pressures, a provider will take “shortcuts” such as reducing the number of diagnoses recorded during a visit, increasing their use of follow-up care (i.e., pushing care onto future visits), and providing less preventive care. Similarly, Neprash and Barnett [58] found that providers were more likely to prescribe opiates later in their shifts, and Chan [59] found that emergency room physicians spent less time with patients near the end of their shift, resulting in higher total costs because these patients used more hospital resources. The provider’s decisions may be rational to them, but they create inefficiencies for the organization. Eliminating inefficiencies stemming from suboptimal decision-making may involve developing and testing strategies that have emerged from the field of behavioral economics, such as nudges and clinical reminders [60, 61]. Behavioral economics is a newer area of research, and there is much to be learned about how to best adapt nudges for specific contexts and avoid unintended problems like information overload or employee burnout.

### Payment and financing systems

Different payment and reimbursement mechanisms will alter the incentives for improving efficiency. In the U.S., prospective payment was introduced as a way to encourage efficiency by incentivizing providers to minimize the cost of the care they produced, but hospitals responded by providing more intensive services of questionable value that received higher reimbursement [62]. Capitated payment, a payment mechanism in which health care systems are paid a fixed amount per member per month, encourages efficiencies, at the risk of limiting appropriate services [63]. Implementation scientists should consider financing when suggesting strategies for improving efficiency because organizational leaders may not be receptive, especially if the strategy seeks to reduce care that is profitable to the organization. Ultimately, if the payment system does not incentivize allocative efficiency, policy change may be necessary [64].

## Efficient implementation and sustainability

Health care markets do not provide health care organizations with signals on where they need to improve. Instead, that is the vision for creating learning health care systems [2, 65], wherein implementation science fulfills a critical role of helping organizations improve their productive and allocative efficiency. A likely challenge for implementation science is developing methods for assessing efficiency more continuously. A recent study [66] provided insights on what this might look like in the future.

Pluta and colleagues [66] describe an implementation trial that tested the efficient deployment of strategies to improve the impact of smoking cessation, measured in terms of the number of smokers reached and the number of smokers who quit [66]. In brief, the U.S. National Cancer Institute (NCI) funded 52 NCI-designed cancer centers through the Cancer Center Cessation Initiative (C3I) to increase smoking cessation in oncology settings. The centers were allowed to invest the funding into different smoking cessation programs. They could invest in programs that had a greater reach, though these programs were typically less effective. Alternatively, they could invest in more intensive programs, such as those employing in-person counseling, which is typically more effective but reaches fewer people. Pluta and colleagues [66] measured efficiency with an output maximization model using DEA. The results showed wide variability across sites in terms of their reach and effectiveness, as well as variation in the resources each site used. They highlight one site as being most efficient in terms of using its resources to reach the greatest number of people while also generating the highest number of patients who successfully quit smoking.

Efficiency analysis, whether based on DEA or a stochastic frontier analysis, has the potential for each site to learn from themselves and from each other. Sites may reflect on the degree to which their selected strategies are operating efficiently, as indicated by being on the frontier of Fig. 2, or inefficiently. Inefficient sites could seek ways to reduce their costs and become more efficient. In this never-ending quest to be more efficient, each site will need to decide when to stop pursuing one strategy and pivot to another. If the site’s smoking cessation program is operating on the flat of the curve, the program could stop and pivot. Of course, there are no easy litmus tests to guide these decisions, but efficiency is essential to sustaining evidence-based practices.

Measuring efficiency using a DEA or stochastic frontier model has advantages over alternative approaches that necessitate creating and updating a CEA model [67]. One option is to assess outputs and inputs at regular intervals, allowing the site to learn by comparing this month to prior months and by comparing themselves to their peers. Imagine the study conducted by Pluta et al., where the implementation costs and implementation outcomes were collected monthly. Sites could use this information to make improvements and guide their implementation strategy. If reach declined, the team could consider investing in alternatives strategies or focus on a different outcome, like effectiveness. The main limitation, however, with these efficiency models is causal identification. [68, 69] “Efficient” sites may appear inefficient if the analytical model is incorrectly specified (e.g., an omitted variable). This may be mitigated by following organizations over time with machine learning techniques that can model efficient production more accurately [70].

## Conclusion

Health care leaders often rely on economic evaluations for insights. An organizational leader may say they want a CEA, but if they really want to know whether an implementation strategy will save them money in the next year, then the CEA’s focus on allocative efficiency is unlikely to address the leader’s specific question. Instead, a budget impact analysis, focused on productive efficiency will be more likely to address that question. Because CEA and BIA address different aspects of efficiency, these tools are not interchangeable. Therefore, implementation scientists should seek clarity when designing economic evaluations to ensure their methods address the underlying question.

As described earlier, economic evaluations can be achieved using decision models and observational data analyses. Both methods have strengths and weaknesses that make them complimentary. Decision models require information on chance probabilities and costs, which may come from the literature or national databases, and may not be relevant to an organization faced with a decision. Similarly, showing that investments in telehealth reduce ED visits with a regression model may be relevant to those organizations in the sample, but the results may not generalize to organizations outside the sample. Both methods require specialized skills and differentiating the two methods may help implementation scientists who are seeking collaborators, a well-known challenge [71, 72]. Implementation scientists wanting to conduct a CEA may have more success searching for experts in decision sciences and operations research. Conversely, if they interested in measuring budget impact using administrative data, they may find more interest among health economists.

Economic evaluations should include the costs of implementation, which often need to be estimated using micro-costing methods. When estimating implementation costs, researchers need to decide how precisely to estimate costs and the unit of analysis. More precision incurs substantially higher measurement burden [73, 74]. In addition, in economic evaluations where the patient is the unit of analysis, researchers will need to identify the number of patients who were exposed to the implementation strategy. This may require further data collection efforts. Productive efficiency models, on the other hand, often focus on the site or clinic as the unit of analysis, and this reduces the micro-costing burden.

In health care, like other markets that do not exhibit perfect competition, efficiency gains may raise questions about equity. As an example, a narrow focus on maximizing the number of patients screened for colorectal cancer (or minimizing the cost of reaching a particular number of patients eligible for colorectal cancer screening), may yield strategies that exacerbate health inequities by focusing on the easiest to reach patients, leaving patients from marginalized populations at a disadvantage. Thus, the issue of equity and fairness cannot be easily avoided when seeking to improve efficiencies [75]. This phenomenon can be countered by explicitly identifying equity as a goal. For example, distributional CEA explicitly includes equity [76], and examining treatment effect heterogeneity can inform equity questions in observational analysis [77].

This paper is not meant to be an exhaustive review of efficiency theory and methods. Readers interested in deeper discussions on theory and measurement will likely find Sickles and Zelenyuk’s [17] and Greene’s [78] books of interest. There are also important limitations when conducting efficiency analysis, especially with small samples and causal identification, so efficiency analysis should not be viewed as a universal solution.

In summary, implementation science has developed as a robust interdisciplinary field. While there are challenges to implementation scientists collaborating with health economists and decision scientists, a common understanding of efficiency concepts and methods can help bridge existing gaps. Health economists and decision scientists have several powerful tools that may be useful, but these tools are not interchangeable, in part because they are often viewing efficiency problems through a different lens. We believe a greater understanding of efficiency will help focus research designs and help support ongoing collaborations.

## Data Availability

Not applicable.
